# Use of modified ichip for the cultivation of thermo-tolerant microorganisms from the hot spring

**DOI:** 10.1186/s12866-023-02803-2

**Published:** 2023-03-03

**Authors:** Juntian Zhao, Yasmeen Shakir, Yulin Deng, Ying Zhang

**Affiliations:** 1 School of Life Science, Beijing Institute of Technology, Beijing, 100081 China; 2 Department of Biochemistry, Hazara University, Mansehra, Pakistan

**Keywords:** Thermo-tolerant microorganism, Ichip, Tengchong hot spring, Uncultured microorganisms

## Abstract

**Background:**

Thermostable microorganisms are extremophiles. They have a special genetic background and metabolic pathway and can produce a variety of enzymes and other active substances with special functions. Most thermo-tolerant microorganisms from environmental samples have resisted cultivation on artificial growth media. Therefore, it is of great significance to isolate more thermo-tolerant microorganisms and study their characteristics to explore the origin of life and exploit more thermo-tolerant enzymes. Tengchong hot spring in Yunnan contains a lot of thermo-tolerant microbial resources because of its perennial high temperature. The ichip method was developed by D. Nichols in 2010 and can be used to isolate so-called “uncultivable” microorganisms from different environments. Here, we describe the first application of modified ichip to isolate thermo-tolerant bacteria from hot springs.

**Results:**

In this study, 133 strains of bacteria belonging to 19 genera were obtained. 107 strains of bacteria in 17 genera were isolated by modified ichip, and 26 strains of bacteria in 6 genera were isolated by direct plating methods. 25 strains are previously uncultured, 20 of which can only be cultivated after being domesticated by ichip. Two strains of previously unculturable Lysobacter sp., which can withstand 85 °C, were isolated for the first time. Alkalihalobacillus, Lysobacter and Agromyces genera were first found to have 85 °C tolerance.

**Conclusion:**

Our results indicate that the modified ichip approach can be successfully applied in a hot spring environment.

**Supplementary Information:**

The online version contains supplementary material available at 10.1186/s12866-023-02803-2.

## Introduction

As a precious high-temperature extreme environment on the earth, hot springs are rich in thermophilic and thermo-tolerant microbial resources. In this extreme environment, microorganisms catalyze many critical biological reactions, maintaining environmental stability and self-growth [1]. This kind of microorganism lives in a high-temperature environment. Its cells and enzyme proteins have exceptional heat resistance so thatthey can be developed and utilized as important microbial resources with broad development space. Thermo-tolerant microorganisms have been widely used in the food brewing industry, fermentation industry [2], biological fertilizer preparation [3], environmental protection and other fields. For example, inoculating thermophilic and thermophilic microorganisms into agricultural wastes to prepare biological fertilizers has improved the maturity rate and the quality of biological fertilizers [3]. Tengchong is located at the junction of the Eurasian plate and the Indian plate. Intense tectonic activities occurred in geological history, leading to multi-volcanoes and multi-hot springs in the Tengchong area [4]. The Rehai Geothermal Field, located in Tengchong County, is China’s largest and most intensively studied geothermal field. A wide physicochemical diversity of springs (ambient to ~ 97 °C; pH from ≤1.8 to ≥9.3) provides a multitude of niches for extremophilic microorganisms [5–7]. Therefore, cultivating microorganisms in hot springs in Tengchong, Yunnan Province, in the laboratory will provide people with many precious extreme microbial resources.

The secondary metabolites of microorganisms are the main substances available to human biological resources because they can provide raw materials for developing new drugs [8]. For example, the mining of antibiotics [9, 10] and enzymes is based on microorganisms. Especially when looking for new antibiotics or new molecules with medicinal value, microbes in extreme environments can provide us with a unique molecular basis for drugs [11].

With the development of molecular biology, people have developed methods for analyzing environmental microbial communities independent of culture [12], and more unculturable microorganisms have been found [13]. High-throughput sequencing technology can help us analyze the microbial diversity of hot spring samples more comprehensively at the molecular level [14]. However, the pure culture of these microorganisms cannot be obtained [15], and these microorganisms cannot be further utilized. One of the biggest challenges for complex microbial communities is to isolate and cultivate unculturable species, which is the basis of community function and reconstruction [16]. Unculturable and low-abundance species generally exist in different environmental communities; hot springs are no exception, and studying them may lead to important discoveries in life science.

According to the statistics, less than 3% of the total environmental microorganisms can be cultured in the laboratory [17]. These microorganisms cannot be cultured in the laboratory because people lack an understanding of microbial growth conditions and cannot meet the conditions needed for microbial growth [18]. Past experiments have proved that in situ culture and co-culture are the most commonly used methods to study unculturable microorganisms in the laboratory [19]. In situ culture can provide the necessary nutrients for microbial growth and meet the growth conditions of microorganisms by using the original living environment or the original living environment simulated in the laboratory without knowing the growth conditions of microorganisms [20]. The co-cultivation method draws lessons from the survival mechanism of the natural microbial community and uses the interaction between microorganisms to promote the growth of previously unculturable microorganisms [15, 21]. In summary, on this basis, developing some efficient new methods of microbial culture is very important to obtain microbial germplasm resources from hot springs.

The development and utilization of culturable microbial resources are coming to an end, and it is urgent to excavate the original un-culturable microbial resources [22]. To realize the laboratory culture of unculturable microorganisms, in 2002, the Kaeberlein team designed and used a “diffusion chamber” device to analyze the microbial composition in marine sediments [23]. Based on the diffusion chamber, the team optimized the diffusion chamber through continuous exploration and developed ichip in 2010. They moved cultivation into the microbes’ natural habitat by placing cells from varying environmental samples into diffusion chambers, which were then returned to the natural environment for incubation. The design principle of ichip perfectly used in situ culture method in a high-throughput platform. The ichip composed of several hundred miniature diffusion chambers, each inoculated with a single environmental cell. The team demonstrated that cultivation of environmental microorganisms inside the ichip incubated in situ leads to a significantly increased colony count over that observed on synthetic media. It is reported that the ichip is far superior to standard cultivation in the aspect of isolation of soil and sediment microbial samples, and the grown species are of significant phylogenetic novelty [10, 19, 24]. The new method allows access to a large and diverse array of previously inaccessible microorganisms, however, whether the two chips are suitable for extreme water environment samples such as hot springs has not been reported.

Here, we describe the first attempt using ichip to isolate cultivable and previously uncultivated bacteria from Tengchong hot spring. To make ichip more suitable for use in hot water environments up to 90 °C, we have modified the design of ichip. The overall working principle of the modified chip is the same as that of ichip. We also compared the isolation efficiency of direct plating methods with that of ichip. Meanwhile, the MiSeq method was used to understand the microbial community structure in the sample comprehensively.

## Materials and methods

### Sample collection and treatment

The sample of this study is taken from the DaGunGuo in Rehai, Tengchong, Yunnan, China. The GPS location is 24.95355 ° N, 98.43812 ° E, and the temperature is about 90 °C. After the samples are collected, they are stored in a thermos and transported to the laboratory within 2 days.

**Fig. 1:**
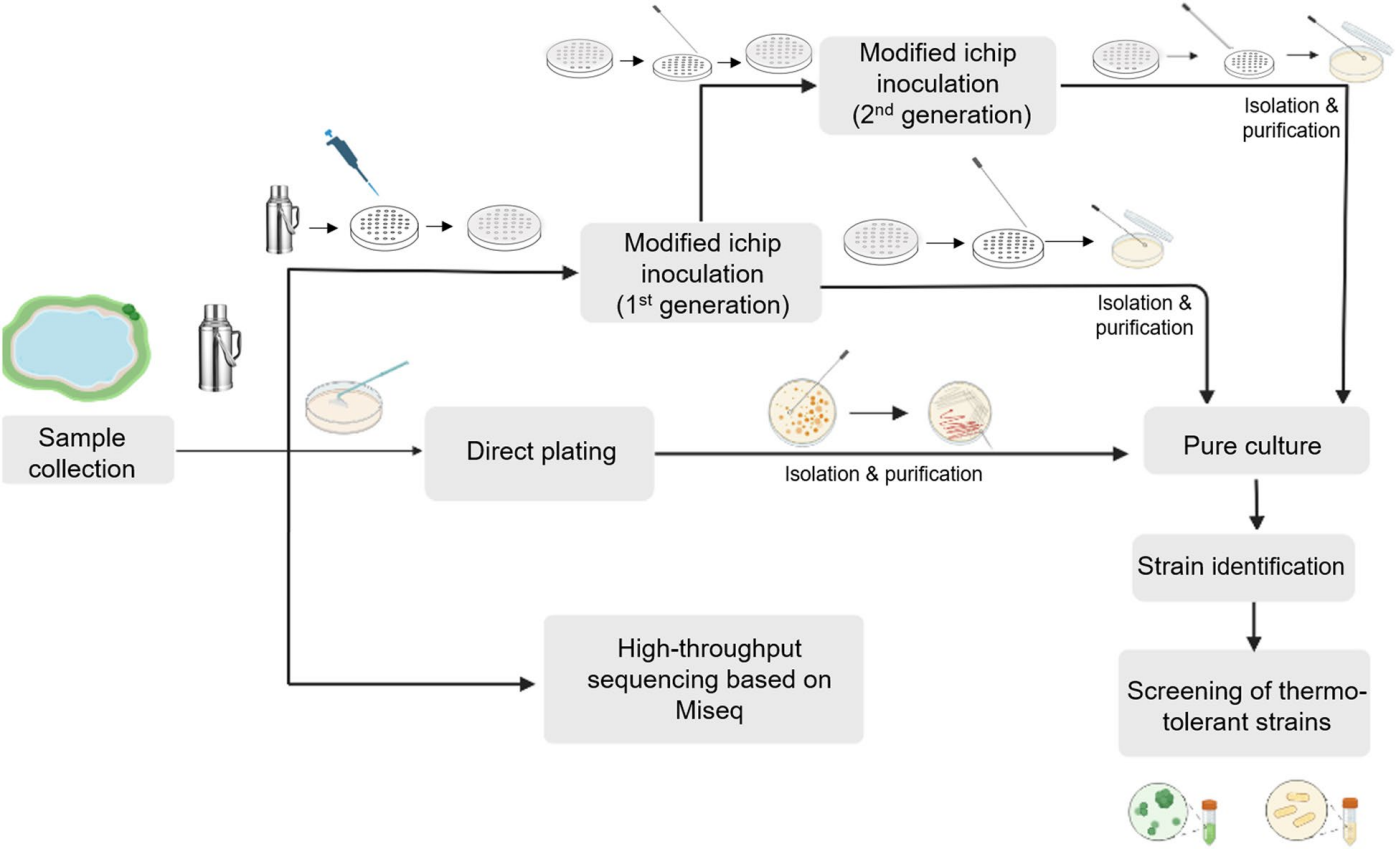
Flow-chart of the entire experiment process

Immediately after the sample arrived in the laboratory, the following three parts of experiments were carried out at the same time. (1) The sample was inoculated into modified ichips, the inoculated chips were placed at 85 °C for 8 weeks as the first domestication experiment. (2) Direct plating method was used to isolate microorganisms in hot spring water. (3) The genomic DNA of microorganisms was directly extracted from hot spring water and then Miseq high-throughput sequencing analysis was carried ou. The whole experimental process is shown in Fig. 1.

### Modification of ichip

**Fig. 2:**
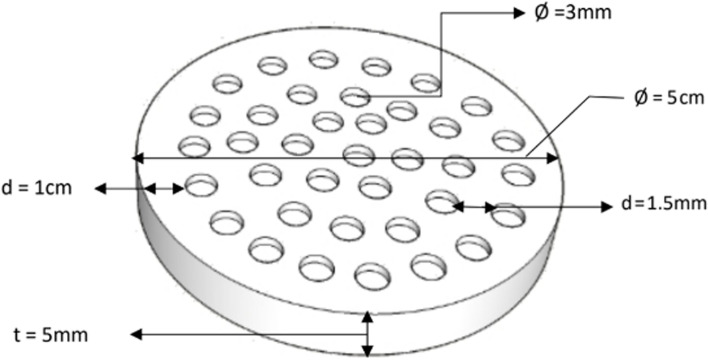
The dimensions of modified ichip

The microbial culture chip used in this experiment has the same design principle as the chip reported by D. Nichols in 2010 [24]. To make the ichip more suitable for analyzing hot spring water samples, we made three major changes to ichip. First, we slightly changed the shape and pore size of the chip. Second, we replaced the agar in the hole with gellan gum [25]. Third, we removed the upper and bottom plates used to fix the membrane in the ichip structure and directly adhered the membrane to the central (loaded) plate with glue [19]. The specific information is described as follows. The modified ichip material used in the experiment is polypropylene plastic, with a thickness 5 mm, diameter 5 cm, and inner diameter 3 cm, there are 37 holes in the middle of the modified ichip, and there are no holes in the edge of the peripheral 1 cm of the chip, so that the film can be fixed with glue. The diameter of each hole is 3 mm, and the distance between the holes is 4.5 mm, which is used to fix the membrane with glue so that each hole is separated from the other and used as a separate microbial culture chamber to culture microorganisms. Figure 2 shows the dimensions of the modified ichip.

### First-generation in situ culture with modified ichip

**Fig. 3:**
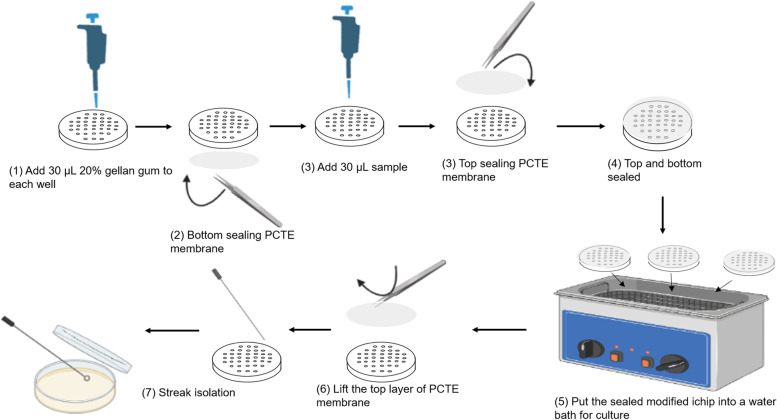
The operation steps of modified ichip culture

To ensure the aseptic operation, modified ichips and 20% gellan gum (Sigma-Aldrich, China, P81691006) were autoclaved in advance, and the whole assembly process was completed on the clean bench. The specific operation of modified ichip culture is as follows: (1) 30 μL of 20% gellan gum (Sigma-Aldrich, China, P81691006) was added to each hole of modified ichip; (2) after the solidification of gellan gum, RTV 108 glue (Momentive, USA,6502883876) was applied to seal the bottom of modified ichip with a PCTE membrane (GVS, USA,1239558) with a pore diameter of 0.03 μ m; (3) later, a micropipette was used to absorb 10 μ L hot spring samples and inoculate them into each hole of modified ichip; (4) then,the top of the modified ichip was sealed with a PCTE membrane with a pore diameter of 0.03 μ m; (5) then 5 L hot spring water was poured into the water bath. At the same time, a blank control was prepared (chip without inoculation). All the prepared modified ichips were placed into the water bath pot, and the modified ichip remained floating. The specific steps are shown in Fig. 3. The hot spring water in the water bath was replenished once a week to ensure that the microbes in the modified ichip have enough nutrients. Nutrients spread from the bottom of the modified ichip, and microbes can grow in each hole. The temperature of the water bath was set to 85 °C to simulate the original living environment of hot spring microorganisms. After incubating at 85 °C for 8 weeks, the membrane on the upper side of the modified ichip was carefully opened. The microorganisms in each hole were marked on R2A Agar [26] (Coolabar, China) (consisting of yeast extract fermentation 0.5 g, peptone 0.5 g, lactium 0.5 g, glucose 0.5 g, solublestarch 0.5 g, dipotassium hydrogenphosphate 0.3 g, magnesium sulfate 0.024 g, sodium pyruvate 0.3 g, agar 15 g and distilled water 1 L) and FW70 medium [27] (consisting of sodium pyruvate 1 g, casamino acids 1 g, gellan gum 15 g and hot spring water 1 L) with toothpicks (aseptic), cultured at 37 °C for 72 h, which were observed every 24 hours; after 72 h the step of isolation and purification of single colonies was performed. Finally, each strain’s phylogenetic status was identified by amplifying 16S rRNA gene sequence analysis [28], and the identification method is described in detail in the Strain identification section. Because this operation step is to put microorganisms into the ichip for in situ culture for the first round, we termed it “the first generation *in situ* culture”.

### Second-generation in situ culture with modified ichip

**Fig. 4:**
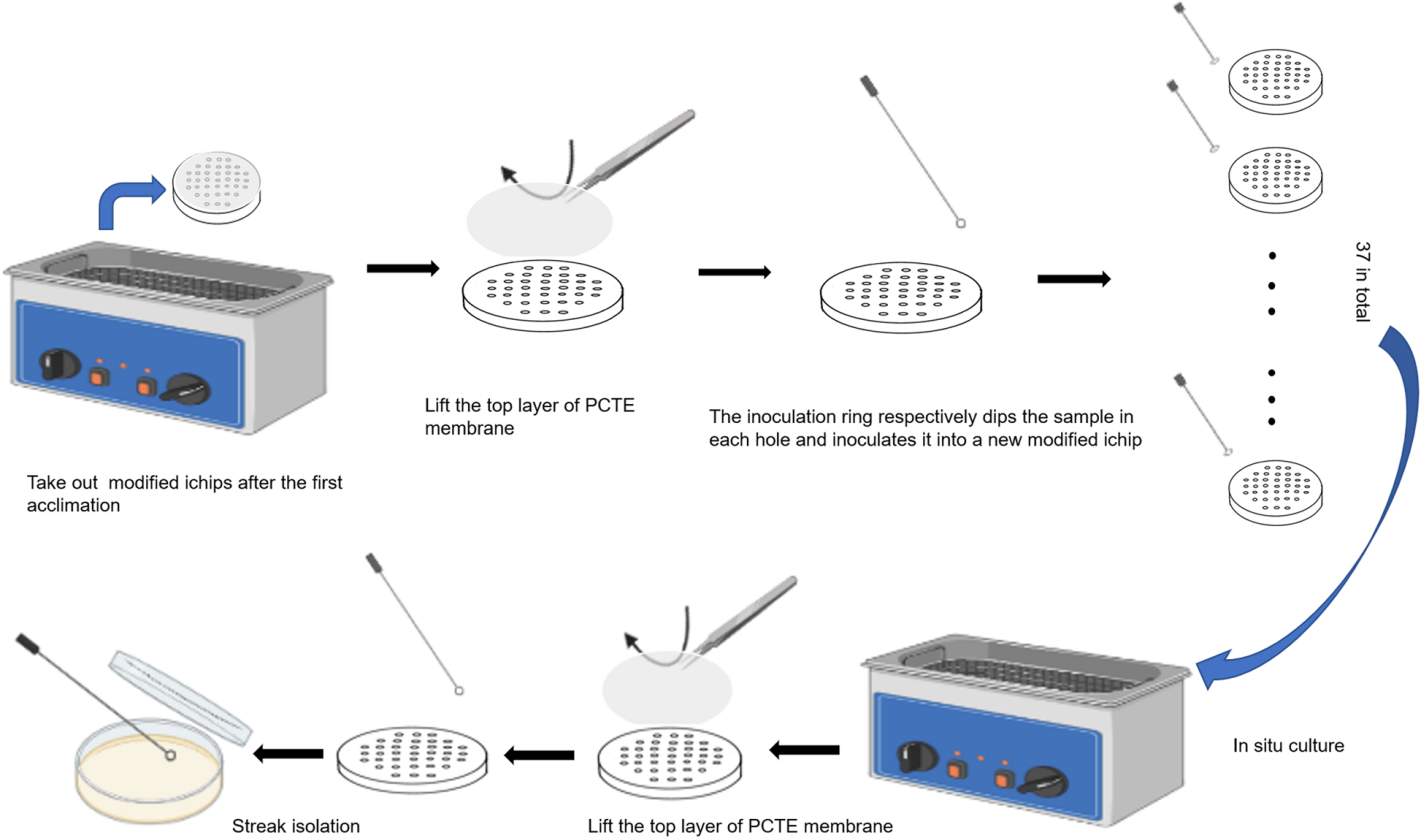
Modified ichip transfer culture operation method

Two of the four modified ichips cultured in situ were selected for direct line isolation, purification and identification. The remaining two were transferred into the new modified ichips for in situ culture for 30 days. Because this operation step is to put microorganisms into the ichip for in situ culture for the second round, we termed it “the second generation *in situ* culture”. The specific method is as follows: After the first domestication, open the upper membrane of ichip. The inoculation needle was used to transfer the cultured products in one hole of the first generation of ichip 37 times into 37 holes of a newly prepared ichip, the new modified ichips were placed into a water bath for a second in situ culture, as shown in Fig. 4**.** After 1 month of incubation, the samples in each well of modified ichip were isolated and cultured on an R2A plate, respectively, and the isolated microorganisms were identified. The purpose of this procedure was further to verify the isolation efficiency of modified ichip for samples.

### Isolation and purification with direct plating methods

The hot spring water samples were directly inoculated on R2A Agar and FW70 medium, cultured at 37 °C, and observed every 24 h. The microorganisms were further isolated and purified under the same culture conditions and the 16S rRNA amplification was performed to identify the bacterial strains.

### High-throughput sequencing based on Miseq

The hot spring water samples were filtered with an MF-Millipore membrane filter (Millipore, Germany, GSWP02500) with a pore diameter of 0.22 μm [29]. The microorganisms intercepted on the filter membrane were fully eluted with normal saline. Genomic DNA was extracted by EZNA soil DNA extraction kit (Omega Biotek, Doraville, GA, USA, D562501), and the V3V4 [30] region of the sample was sequenced by the Miseq method adopted by Allwegene (Allwegene, Beijing, China) Company.

### Screening of thermo-tolerant strains

Five different temperatures were set, i.e., 45 °C, 55 °C, 65 °C, 75 °C, 85 °C [31–33], and all the strains obtained by direct plating methods and modified ichip were inoculated into 6 ml R2A liquid medium. For each strain, 15 tubes were inoculated, and 3 tubes were placed at each temperature as a repeated test. The growth of bacteria was observed after 72 h liquid culture at 100 rpm.

### Strain identification

The streak plate technique was employed to obtain the pure culture, and then the microbial classification was carried out according to the colony morphology. 16S rRNA gene sequence was used for the identification of bacterial strains. TIANamp Bacteria DNA Kit (China, TIANGEN) was used to extract the DNA. The extracted DNA of bacteria was used as the template to carry out the polymerase chain reaction (PCR), and P0 and P6 [34] (Table 1) were used as primers to amplify the 16S rRNA gene of bacteria. The total PCR reaction mixture was 25 μ L, i.e., 10× Taq Buffer 2.5 μ L, dNTP Mixture (2.5 mmol/L) 0.5 μ L, MgCl_2_ 1.5 μ L, primer P0 0.5 μ L, primer P6 0.5 μ L, Taq enzyme (5 U / μ L) 0.3 μ L, template (total genomic DNA) 2 μ L, sterile water 17.2 μ L. The PCR amplification conditions were as follows:

$$95\;^\circ\mathrm C\;\left(1\;\min\;30\;\mathrm s\right)\;-\;\left[95\;\;^\circ\mathrm C\;\left(1\;\min\;30\;\mathrm s\right)\;-\;56\;^\circ\mathrm C\;\left(30\;\mathrm s\right)\;-72\;^\circ\mathrm C\;\left(1\;\min\;30\;\mathrm s\right)\right]\;\left(25\;\mathrm{cycles}\right)\;-\;72\;^\circ\mathrm C\;\left(10\;\min\right)\;-\;4\;^\circ\mathrm C$$

**Table 1:** Primers sequence

Primers	Seqence
P0	5′-GAGAGTTTGATCCTGGCTCAG-3’
P6	5′-CTACGGCTACCT TGTTACGA-3’

The full-length sequence of 16S rRNA gene was sequenced by pyrophosphate sequencing and spliced. In the analysis and comparison of the microbial sequence, only those with 100% consistent sequence are considered to be the same microorganism. To identify the microbial species, the full-length base sequences of 16S rRNA gene were compared in the NCBI database. Researchers generally believe that the similarity of bacterial 16S rRNA below 98.7% can be considered a new bacterial species, and less than 94.50% can be considered a new bacterial genus [35].

The 16S rRNA sequences of all strains obtained were uploaded to NCBI GenBank, and the registration numbers are listed in the Supplementary Information Tables.

## Result

To verify the completeness of the chip’s seal, we made empty chips using the following methods. We loaded the chips with sterile gellan gum and assembled the membrane without inoculation. We incubated them in LB (Luria-Bertani) medium. After incubation, we observed no growth inside the chips. In the second set of experiments, we loaded the chips with sterile gellan gum, assembled the membrane after *E. coli* and *Rhodococcus* sp. cells inoculation, and incubated the assembled chips in a sterile LB medium. After incubation, we observed two types of strains’ growth inside the chips. And no growth was observed outside the chips. This proved that the seal provided by RTV 108 glue was sufficient to prevent bacteria cells from migrating in and out of chips. As a control experiment for field experiments, we also incubated the empty chip in hot spring water with other inoculated chips; no growth was observed inside the chips.

This study used ichip, direct plating methods, and high-throughput sequencing to evaluate all microorganisms in Tengchong hot spring water. For ichip, we have compared the first-generation in situ culture and the second in situ culture after transfer. Since most previous studies on thermo-tolerant microorganisms were reported at the genus level, and the sequencing results obtained by Miseq and 16S rRNA full-length pyrosequencing were more reliable at the genus level, so we analyzed our result at the genus level.

### Microbial isolation after the first generation in situ culture with modified ichip

After the inoculated modified ichip was simulated in situ culture in the laboratory, the bacteria in the chip were isolated and purified on FW70 and R2A Agar. After 16S rRNA gene identification, 47 strains of bacteria and 12 genera were obtained. The 12 genera are *Agromyces, Alkalihalobacillus, Pannonibacter, Pseudomonas, Bacillus*, *Lysobacter, Actinotalea, Bosea, Hyphomicrobium, Kocuria, Microbacterium, Sphingomonas.* It was found that the number of *Agromyces, Alkalihalobacillus, Pannonibacter, Pseudomonas, Bacillus*, uncultured *Lysobacter, Actinotalea* is relatively large among the 12 genera. All the strains obtained are listed in Tab S1. 2 of the 47 strains had less than 98.7% similarity with the 16S rRNA full length in the NCBI database, proving they were new species.

### Microbial isolation after the second generation in situ culture with modified ichip

Two of the four modified ichips cultured in situ were selected and transferred according to the method described in the First generation in situ culture with modified ichip and then cultured in situ again. After the second in situ culture, 60 strains of bacteria were isolated and purified on an R2A plate. Among them, *Bacillus*, *Agromyces*, *Paenibacillu, Pseudomonas, Sphingomonas,* uncultured *Aquiflexum* are abundant, and all the strains obtained have been shown in Tab S2. 8 of the 60 strains had less than 98.7% similarity with the 16S rRNA full length in the NCBI database, which proved that they were new species.

By comparing the difference in isolation efficiency between modified ichip first generation and second generation domestication, the results showed that the number of *Bacillus, Pseudomonas, Sphingomonas* strains increased after second generation domestication with modified ichip in R2A medium. To sum up, second generation domestication of microorganisms cultured with modified ichip significantly improved the isolation efficiency of the microorganisms mentioned above. At the same time, compared with the isolation efficiency of the first generation of modified ichip in situ culture, more than 5 genera were isolated by second generation in situ culture, which were not isolated by first generation in situ culture, namely *Brevibacillus, Brucella, Ochrobactrum, Paenibacillus,* and uncultured *Aquiflexum*. 60 strains of bacteria were obtained by the second generation domestication with modified ichip, while only 30 strains of bacteria were obtained by first generation domestication with modified ichip. It is worth noting that the once unculturable microorganisms reported by Hegazi et al. in 2017 [36] and Li et al. in 2016 [37] were obtained with modified ichip, which were previously uncultured *Lysobacter* and *Aquiflexum.* This shows that increasing the domestication generation and prolonging the domestication time beneficial for obtaining more unculturable microorganisms.

### Isolation and purification by direct plating methods

The hot spring water was directly inoculated on R2A and FW70 solid medium, and 26 strains and 6 genera of bacteria were isolated and purified. By 16S rRNA gene sequencing and comparison, it was revealed that 2 strains belong to *Agromyces*, 3 to *Bacillus*, 13 to *Brevibacterium*, 3 to *Geobacillus*, 1 to *Paenibacillus* and 4 to *Sphingomonas*. The results showed that *Brevibacterium*, *Bacillus, and Sphingomonas* were the dominant bacteria in the hot spring samples. All strains obtained are listed in Tab S3. 5 of the 26 strains had less than 98.7% similarity with the 16S rRNA full length in the NCBI database, proving they were new species.

### Screening of thermo-tolerant strains

76 of the 133 strains obtained by direct plating methods and modified ichip culture were thermo-tolerant strains summarized in Tab S4. Among them, there are 11 strains with the highest temperature tolerance of 45 °C, 42 strains with the highest temperature tolerance of 55 °C, 15 strains with the highest temperature tolerance of 65 °C, 2 strains with the highest temperature tolerance of 75 °C, and 6 strains with the highest temperature tolerance of 85 °C. In conclusion, previous reports have not reported that the strains of *Alkalihalobacillus, Lysobacter* and *Agromyces* can tolerate high temperatures of 85 °C. *Alkalihalobacillus* and *Lysobacter* were all isolated by modified ichip and could not be isolated by direct plating methods. And the *Lysobacter* strain was isolated for the first time in this study, which was once unculturable.

### High-throughput sequencing based on Miseq

The results of high-throughput sequencing showed that there were at least 29 genera of bacteria in the sample, of which *Bacillus* accounted for the 45.2%, followed by *Pannonibacter* 21.07%， *Hydrogenobacter* 8.11%, *Nitriliruptor* 7.84%, *Brevibacterium* 6.68%, *Bacteroides* 2.05%, *Akkermansia* 1.64%, *Paenibacillus* 1.13%, *Alicycliphilus* 0.74%, Others 5.54%.

### Comparison of high-throughput sequencing, modified ichip, and direct plating methods

**Fig. 5:**
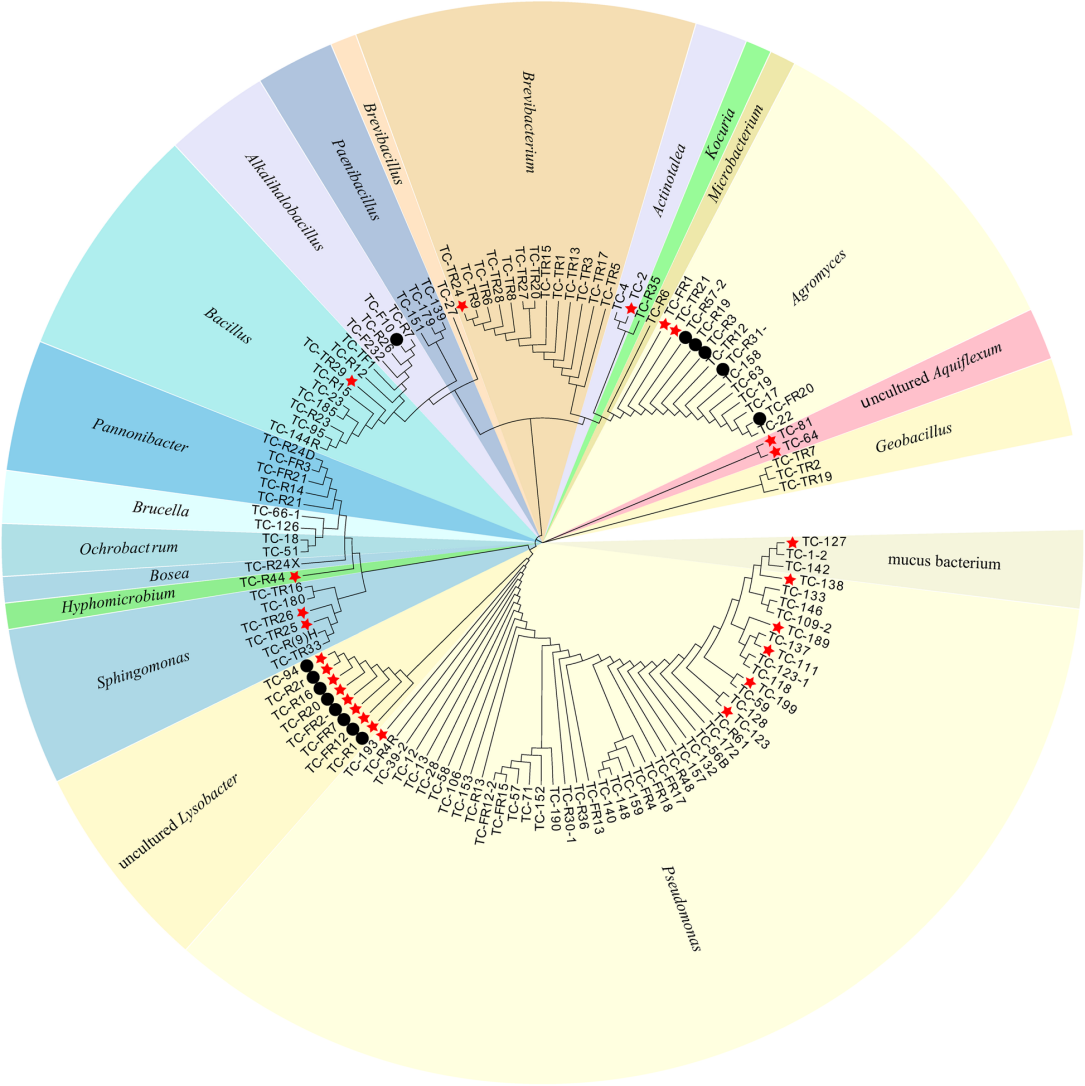
Phylogenetic tree of 16S rRNA genes showing the position of all isolated strains. The tree was constructed using the Neighbor-Joining method and Kimura’s 2-parameter model. Bootstrap values (obtained with 1000 sub-samples) > 50% are indicated at the nodes. (The red star in the figure represents uncultured strains, and the black dot represents the first obtained heat-resistant strains)

The phylogenetic status of these 133 strains is shown in Fig. 5, and the statistical analysis is given in Table 2. The results show that modified ichip domestication can significantly improve the isolation ability of hot spring microorganisms, which is reflected in two aspects: on the one hand, for the genera that can be isolated from direct plating methods, modified ichip domestication can obtain more strains. For example, more than 4 strains of *Agromyces* and 4 strains of *Bacillus* were isolated from modified ichip than from direct plating methods. On the other hand, modified ichip can cultivate microbial species that direct plating methods cannot isolate. For example, *Actinotalea, Alkalihalobacillus, Hyphomicrobium, Kocuria, Pannonibacter, Microbacterium, Pseudomonas,* uncultured *Lysobacter* and *Bosea* can only be isolated from ichip, and none of these microorganisms can be isolated by direct plating methods method. Among the 76 strains of thermo-tolerant bacteria obtained, 55 strains were from modified ichip, and 21 strains were from direct plating methods. Modified ichip could obtain more thermo-tolerant microorganisms than direct plating methods.

**Table 2:** Summary of microorganisms of all genera detected by Modified Chip, Direct Plating and Miseq high-throughput sequencing (“-“means not detected)

Genus	Proportion (Miseq high-throughput sequencing) (%)	Direct plating methods	Modified ichip first generation	Modified ichip second generation
*Actinotalea*	–	–	1	–
uncultured *Actinotalea*	–	–	1	–
uncultured *Lysobacter*	–	–	7	1
*Alkalihalobacillus*	–	–	4	–
*Pannonibacter*	21.0689	–	5	–
*Pseudomonas*	–	–	13	26
*Hyphomicrobium*	–	–	1	–
*Bosea*	–	–	1	–
*Microbacterium*	0.0504	–	1	–
*Kocuria*	–	–	1	–
uncultured *Aquiflexum*	–	–	–	2
*Ochrobactrum*	–	–	–	2
*Brucella*	–	–	–	2
*Brevibacillus*	–	–	–	1
*Agromyces*	–	2	6	4
*Bacillus*	45.2028	3	3	5
*Brevibacterium*	6.6772	13	–	–
*Geobacillus*	–	3	–	–
*Paenibacillus*	1.1345	1	–	3
*Sphingomonas*	–	4	1	2
*Alkalicoccus*	0.1152	–	–	–
*Diaphorobacter*	0.0216	–	–	–
*Pajaroellobacter*	0.0108	–	–	–
*Phenylobacterium*	0.4322	–	–	–
*Parabacteroides*	0.2197	–	–	–
*Paracoccus*	0.0612	–	–	–
*Muribaculum*	0.2305	–	–	–
*Akkermansia*	1.6423	–	–	–
*Alicyclobacillus*	0.2917	–	–	–
*Prevotella*	0.0972	–	–	–
*Hydrogenobacter*	8.107	–	–	–
*Egicoccus*	0.6663	–	–	–
*Alloprevotella*	0.4358	–	–	–
*Eubacterium*	0.2161	–	–	–
*Mogibacterium*	0.2089	–	–	–
*Roseburia*	0.2017	–	–	–
*Alicycliphilus*	0.7383	–	–	–
*Alistipes*	0.479	–	–	–
*Rubrobacter*	0.1369	–	–	–
*Nitriliruptor*	7.8441	–	–	–
*Hydrogenophilus*	0.1044	–	–	–
*Rhodococcus*	0.0432	–	–	–
*Bacteroides*	2.0529	–	–	–
*Blautia*	0.5186	–	–	–
Other bacterium	–	–	2	11
Other uncultured bacterium	0.479	–	–	1

**Fig. 6:**
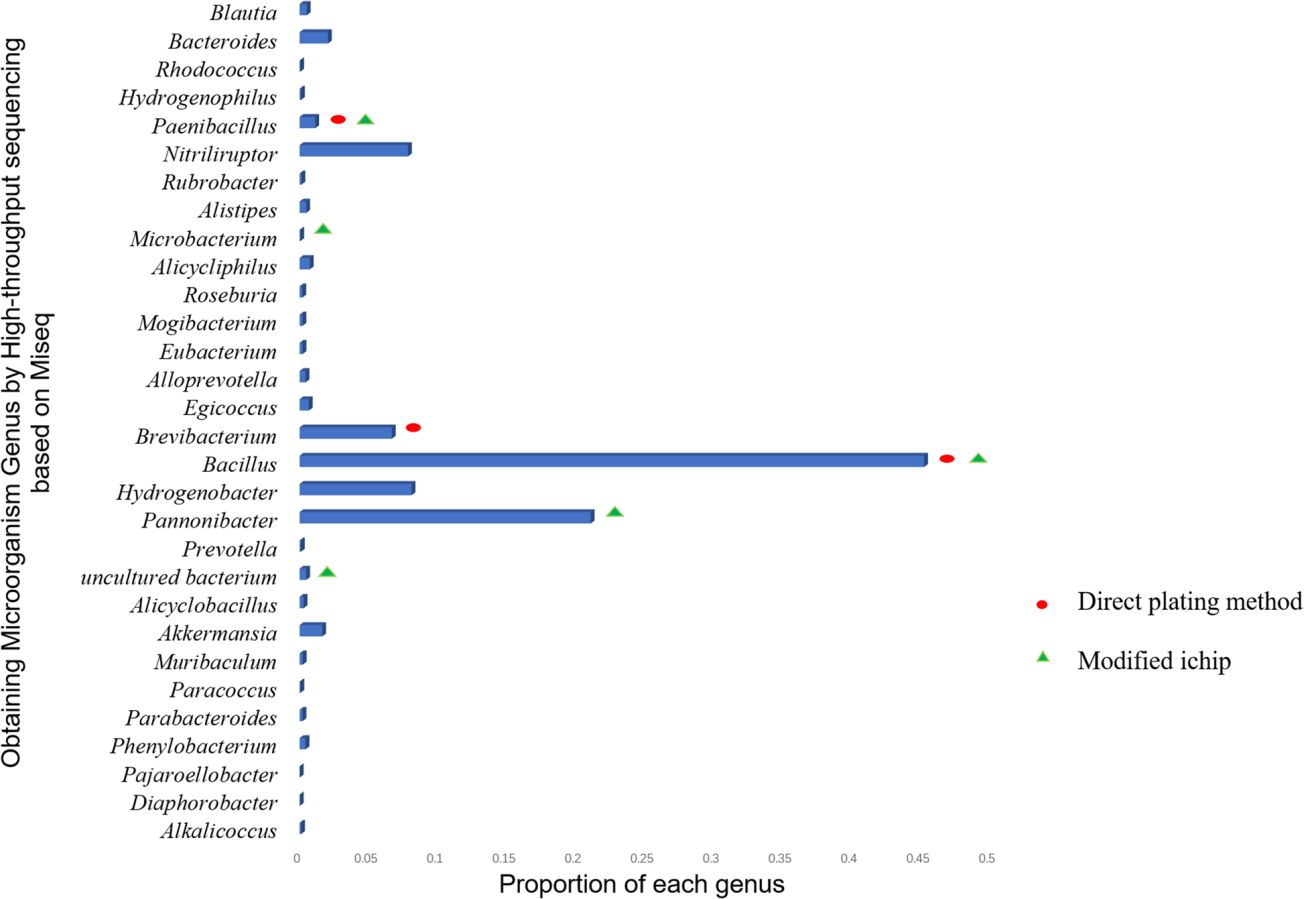
Microbial diversity composition of Tengchong hot spring water based on Miseq sequencing. (The red dot in the figure represents that Direct plating methods can isolate microorganisms of these genera. The green triangle in the figure represents that microorganisms of these genera can be isolated by ichip method. The red dot and the green triangle appear together to represent that microorganisms of these genera can be isolated by both ichip and Direct plating methods)

Fig. 6 shows the proportion of each genus of microorganisms in the sample detected by MiSeq method. The results show that the most dominant bacteria in the hot spring is *Bacillus,* which can be isolated from ichip and direct plating methods. The most dominant bacteria obtained by modified ichip culture are *Pseudomonas*. In addition, the dominant bacteria are *Bacillus, Agromyces* and uncultured *Lysobacter.* Microbes isolated from direct plating methods belong to six different genera. Among them *Brevibacterium, Bacillus, Geobacillus* and *Sphingomonas* were present more frequently, *Agromyces* and *Paenibacillus* were less. *Paenibacillus, Bacillus* and *Brevibacterium* exist in large quantities in the samples detected in the Miseq results. In addition to cultivating strains of high abundance genus, the modified ichip can also obtain strains of low abundance in hot springs, such as *Microbacterium* and some strains that could not be cultured originally.

## Discussion

A hot spring is a typical example of an extremely high-temperature environment where many microbial strains live. These microorganisms can withstand high-temperature environments, and their gene sequences are valuable gene resources. At the same time, the proteins expressed by these microorganisms are heat-resistant proteins, which play a very important role in industrial production and life practice. Currently, the number of microorganisms that can be cultivated by scientific means is less than 3% of the total number of natures, and the number of thermo-tolerant microorganisms. If we want to make better and more extensive use of these thermo-tolerant microbial resources, we must let more thermo-tolerant microorganisms be cultivated in the laboratory. Ichip was invented 13 years ago and widely used in the next decade. For example, in the study of Ling et al. in 2015, the uncultured bacteria that produce new antibiotics were isolated and obtained by ichip [38]. In the study of Berdy et al. in 2017, more oral microorganisms can also be isolated by a miniaturized version of the ichip [19]. Isolation efforts inspired by the ichip were also performed using many kinds of sediments, such as marine sediments, mangrove sediment, and estuarine sediment, etc.. These studies have also helped us obtain many unculturable microorganisms, such as *Actinomycetota* resources and harmful substance degrading bacteria resources [39–41]. Generally speaking, this platform has been shown to increase microbial recovery from 5- to 300-fold, depending on the study [19]. Therefore, we first applied the ichip to domesticate, isolate, and culture thermo-tolerant microorganisms in this study. Verifying whether the ichip can be used for hot spring samples helps us obtain more thermo-tolerant bacteria and originally unculturable microorganisms.

There are two main aspects for the change of the ichip. On the one hand, gellan gum is used to replace the agar because the agar will melt in hot spring water and lose its support for microorganisms. On the other hand, we removed the upper and bottom plates used to fix the membrane in the ichip structure and directly adhered the membrane to the central (loaded) plate with glue. At the same time, the pore diameter of carrying microorganisms is enlarged. This change aims to make the hot spring water easier to contact with microorganisms. The transformation of these two aspects is mainly to make ichip more suitable for hot spring water samples, and there is no change in principle. So our experimental results can reflect the application ability of ichip in analyzing hot spring samples. The experimental results showed that the number and diversity of microbial strains domesticated by ichip were higher than those by direct plating culture. Ichip obtained 20 previously unculturable microorganisms, more than those mentioned in previous studies [42].

Previous studies related to Tengchong hot springs showed that the thermophilic bacteria isolated from Rehai belong to the phyla Firmicutes, Actinobacteria, and Deinococcus-Thermus. Firmicutes include *Anoxybacillus*, *Bacillus*, *Caldalkalibacillus*, *Caldanaerobacter*, *Laceyella*, *Geobacillus*, *Alicyclobacillus* and *Sulfobacillus*. Isolates from the Deinococcus-Thermus phylum include *Meiothermus* and *Thermus*. Several thermoacidophilic archaea belonging to *Acidianus*, *Metallosphaera*, and *Sulfolobus* have also been isolated [43–46]. Traditional culture methods isolated all these microorganisms, but the cultivation methods and conditions used by different researchers are different. On the one hand, it is due to the wide physicochemical diversity of Tengchong hot spring water (10 to 97 °C, pH from ≤1.8 to ≥9.3). For example, some studies used a common salt culture medium, and the pH value was adjusted to neutral; the culture temperature was 70 °C [33]. Some used YIM14 medium, which had basal salt composition designed from the chemical analysis of Dagunguo hot spring water. The pH values ranged from 2.5 to 8.5 and the culture temperature ranged from 20 to 96 °C were used [47–49]. On the other hand, it is due to the different research purposes. The isolated species are mainly dependent on the media. For example, some researchers discovered the reduction of structural Fe (III) in nontronite by thermophilic microbial. The culture conditions they used were anaerobic, NAu-2 stock solution, and lactate served as the sole electron acceptor and donor, respectively. Some strains of genus *thermus* were isolated from the *thermus* medium specially designed for cultivating *thermus* microorganisms [47, 50]. In this study, thermo-tolerant microorganisms are mainly concentrated in 11 genera of microorganisms, including *Bacillus, Agromyces, Alkalihalobacillus, Brevibacterium, Brevibacillus, Geobacillus, Pannonibacter, Pseudomonas, Sphingomonas,* uncultured *Lysobacter, Microbacillus* and *Paenibacillus.* The previous results are partially different from the microbial groups found in this study. The difference may be due to different isolation methods and samples. Our results also indicate that after the domestication of ichip, the species of microorganisms isolated from the R2A medium are also more than that from FW70 media. In previous studies, it has also been reported that the ichip- and Petri-dish-grown collections of microorganisms were markedly different [19].

It is noteworthy that many of the genera we isolated have been reported to have heat resistance ability in other environments, and all these reports used traditional isolation methods. For example, *Bacillus* with thermo-tolerance was found in the soil collected from a commercial edible-oil extraction industry in the report of Saranya et al. in 2014 [51]; *Pannonibacter* with thermo-tolerance was found in a granular activated carbon (GAC) unit currently treating TBA in the study of Reinauer et al. in 2007 [52]; *Microbacteria* with thermo-tolerance were found in a soil sample in Japan identified by Nakata in 2000 [53]; *Brevibacterium* with thermo-tolerance were found in the coastal area of Yellow Sea in South Korea reported by Siddikee et al. in 2010 [54]; Nazina et al. in 2001 reported *Geobacillus* with thermo-tolerance in formation waters of oilfields in Russia [55]; In 2009, Singh reported that *Pseudomonas* with thermo-tolerance were found from loose soil samples from stables in India [56]; *Sphingomonas* with thermo-tolerance were reported by of Eguchi et al. in 2001 in ocean waters near Cape Muroto in Kochi Premium, Japan and the Pacific Ocean [57]. *Brevibacillius* with thermo-tolerance were found in the report of Cotta et al. in 2021 from composing mass [58], *Paenibacillus* with thermo-tolerance were found in the report of Aabed et al. in 2021 from the soil in Saudi Arabia [43], the above are known thermo-tolerant microorganisms, and *Bacillus, Brevibacillus*, *Geobacillus* are common thermo-tolerant microorganisms. *Agromyces, Alkalihalobacillus,* and uncultured *Lysobacter* are the first thermo-tolerant microorganisms found in this study. To some extent, it has been proved that the modified ichip we use has good isolation ability.

Since the direct plating method is used to isolate microorganisms after the domestication of ichip, this research also follows this process. The isolated strains can grow at 37 °C, and some isolated microorganisms fail to withstand high-temperature environments. Even if it is known in the subsequent verification that some microorganisms can withstand high temperatures, they can only be called thermostable microorganisms rather than thermophilic microorganisms. In future research, a high-temperature liquid dilution method can be used to isolate and purify the microorganisms domesticated by the chip, which may be more conducive to obtain more thermophilic microorganisms.

In terms of comparing the microbial isolation efficiency of the chip, theoretically, the High-throughput sequencing technology should be able to detect all the microbial species information in the environmental samples. At the same time, in this study, there are still some microbial strains that the High-throughput sequencing technology cannot detect, and experimental phenomena in this regard are also ubiquitous in the analysis of other environmental samples [59–62]. The reason may be that when genomic DNA of environmental samples is extracted, some microorganisms’ cell wall is challenging to break, resulting in the inability to completely release genomic DNA, which leads to the failure of subsequent molecular analysis.

The existing experimental results can conclude that a modified ichip can obtain more microbial strains with lower abundance in the environment than direct plating culture. More low abundance strains can be obtained by increasing the domestication times and in situ culture time of the modified ichip. It is speculated that the ichip design separates high-abundance microorganisms and low-abundance microorganisms in space for in situ culture, giving microorganisms having low abundance a chance to grow. Due to their slow growth and weak ability to obtain nutrients, some microorganisms in nature may be inhibited by microorganisms with strong growth abilities simultaneously and in space, and these microorganisms form a competitive relationship. The chip weakens or eliminates this competition, increasing the number of low-abundance microorganisms, so they can be isolated later. Several originally unculturable microorganisms were also isolated and purified from the hot spring of Tengchong, Yunnan, China. These microorganisms can grow on an R2A medium during the isolation and purification. Therefore, modified ichip has obvious advantages in cultivating a low abundance of vulnerable bacteria and originally unculturable microorganisms in hot springs.

## Conclusion

1. It was found for the first time that *Agromyces, Alkalihalobacillus* and *Lysobacter* could withstand the high temperature of 85 °C with modified ichip; 5 strains of *Agromyces,* 1 strain of *Alkalihalobacillus*, 8 strains of uncultured *Lysobacter* are thermo-tolerant, which cultivated for the first time by using modified ichip. 15 of the 25 strains of uncultured microorganisms obtained in this study had less than 98.7% similarity with the 16S rRNA full length in the NCBI database, proving they were new species.

2. Our study proves for the first time that modified ichip is suitable for domesticating and isolating microorganisms in hot springs. This method can help us obtain more low-abundance and uncultured microorganisms in hot springs.

## Supplementary Information

**Additional file 1: Tab S1.** Modified ichip culture first generation obtained Strains. **Tab S2.** Modified ichip culture first generation obtained Strains. **Tab S3.** Direct plating methods obtained Strains. **Tab S4.** Screening of thermo-tolerant strains.

## Data Availability

The datasets generated during and zanalyzed during the current study are available from the corresponding author upon reasonable request.
