# Supplementary material for: Use of modified ichip for the cultivation of thermo-tolerant microorganisms from the hot spring
Source: BMC Microbiol. 2023 Mar 3;23:56. doi: 10.1186/s12866-023-02803-2 (PMC9983152; doi:10.1186/s12866-023-02803-2)
Supplement: Supplementary file 1 — Additional file 1: Tab S1. Modified ichip culture first generation obtained Strains. Tab S2. Modified ichip culture first generation obtained Strains. Tab S3. Direct plating methods obtained Strains. Tab S4. Screening of thermo-tolerant strains. [file 12866_2023_2803_MOESM1_ESM.docx]

Use of modified ichip for the cultivation of thermo-tolerant microorganisms from the hot spring

Juntian Zhao^1^·Yasmeen Shakir^2^·Yulin Deng^1^·Ying Zhang^1^ ^*^

***** Ying Zhang

**e-mail:** zhangying3409@bit.edu.cn

1 School of Life Science, Beijing Institute of Technology, Beijing 100081, China

2 Department of Biochemistry, Hazara University, Mansehra, Pakistan

**Tab S1. Modified ichip culture first generation obtained Strains**

| Name of Isolate | Closest Phylogenetic specie | Isolation culture media | 16S rRNA gene similarity (%) | GenBank accession number of isolates |
| --- | --- | --- | --- | --- |
| TC-FR1 | *Agromyces*sp. | FW70 | 98.45% | OP210012 |
| TC-FR12 | uncultured *Lysobacter* sp. | FW70 | 98.82% | ON624173 |
| TC-FR7 | uncultured *Lysobacter* sp. | FW70 | 98.95% | ON624175 |
| TC-FR2- | uncultured *Lysobacter* sp. | FW70 | 99.17% | ON624174 |
| TC-2 | uncultured *Actinotalea* sp. | FW70 | 99.50% | ON624172 |
| TC-F10 | *Alkalihalobacillus halodurans* C-125 | FW70 | 99.51% | ON624162 |
| TC-FR21 | *Pannonibacter phragmitetus* | FW70 | 99.64% | ON624164 |
| TC-4 | *Actinotalea ferrariae* CF5-4 | FW70 | 99.70% | ON624159 |
| TC-FR15 | *Pseudomonas* sp. *WXBSA* | FW70 | 99.86% | ON624166 |
| TC-F232 | *Alkalihalobacillus halodurans* C-125 | FW70 | 99.93% | ON624163 |
| TC-FR12-2 | *Pseudomonas stutzeri* strain W12 | FW70 | 99.93% | ON624168 |
| TC-FR13 | *Pseudomonas stutzeri* strain WWvii23 | FW70 | 99.93% | ON624169 |
| TC-FR20 | *Agromyces* sp. | FW70 | 99.93% | ON624161 |
| TC-FR4 | *Pseudomonas*sp. 2(2013) | FW70 | 99.93% | ON624167 |
| TC-R13 | *Pseudomonas stutzeri* strain QS169 | FW70 | 100.00% | OP076527 |
| TC-R36 | *Pseudomonas stutzeri* strain E141 | FW70 | 100.00% | OP076540 |
| TC-FR3 | *Pannonibacter*sp. | FW70 | 100.00% | ON624165 |
| TC-R44 | *Hyphomicrobium* sp. | R2A | 98.58% | OP076542 |
| TC-R16 | uncultured *Lysobacter* sp. | R2A | 98.83% | OP050440 |
| TC-R2R | uncultured *Lysobacter* sp. | R2A | 99.02% | OP050442 |
| TC-R19 | *Agromyces indicus* | R2A | 99.11% | OP076529 |
| TC-R20 | uncultured *Lysobacter* sp. | R2A | 99.36% | OP050441 |
| TC-R3 | *Agromyces indicus* | R2A | 99.36% | OP076523 |
| TC-R1 | uncultured *Lysobacter* sp. | R2A | 99.45% | OP050443 |
| TC-R61 | *Pseudomonas* sp. | R2A | 99.79% | OP076545 |
| TC-R24D | *Pannonibacter* sp. *strain DJ*-1 | R2A | 99.85% | OP076533 |
| TC-R24X | *Bosea massiliensis* | R2A | 99.85% | OP076532 |
| TC-R15 | *Bacillus* sp. | R2A | 99.91% | OP076521 |
| TC-R57-2 | *Agromyces indicus* | R2A | 99.92% | OP076544 |
| TC-R6 | *Microbacterium aurantiacum* | R2A | 99.92% | OP076525 |
| TC-R21 | *Pannonibacter* sp. | R2A | 99.93% | OP076530 |
| TC-R23 | *Bacillus* sp. CZB12 | R2A | 99.93% | OP076531 |
| TC-R7 | *Alkalihalobacillus halodurans* C-125 | R2A | 99.93% | OP076526 |
| TC-R(9)H | *Sphingomonas* sp. | R2A | 100.00% | OP076522 |
| TC-R12 | *Bacillus halodurans* strain NS928R | R2A | 100.00% | OP076520 |
| TC-R13 | *Pseudomonas stutzeri* strain QS169 | R2A | 100.00% | OP076528 |
| TC-R14 | *Pannonibacter* sp. strain DJ-1 | R2A | 100.00% | OP076528 |
| TC-R26 | *Alkalihalobacillus halodurans* C-125 | R2A | 100.00% | OP076534 |
| TC-R30-1 | *Pseudomonas stutzeri* strain 5-N-1 | R2A | 100.00% | OP076535 |
| TC-R31- | *Agromyces indicus* | R2A | 100.00% | OP076536 |
| TC-R33 | *Bacterium* strain BMV5 | R2A | 100.00% | OP076537 |
| TC-R33-1 | *Bacterium* strain BLEC9.3 | R2A | 100.00% | OP076538 |
| TC-R35 | *Kocuria* sp. AB61 | R2A | 100.00% | OP076539 |
| TC-R36 | *Pseudomonas stutzeri* strain E141 | R2A | 100.00% | OP076540 |
| TC-R42 | *Pseudomonas* sp. strain DKF | R2A | 100.00% | OP076541 |
| TC-R48 | *Pseudomonas stutzeri* strain SM12 | R2A | 100.00% | OP076543 |
| TC-R4R | *Pseudomonas stutzeri* strain FN9 | R2A | 100.00% | OP076524 |

**Tab S2. Modified ichip culture second generation obtained Strains**

| Name of Isolate | Closest Phylogenetic specie | 16S rRNA gene similarity (%) | GenBank accession number of isolates |
| --- | --- | --- | --- |
| TC-106 | *Pseudomonas stutzeri strain* PST-03 | 99.86% | OP087545 |
| TC-107 | *Sphingomonas turrisvirgatae* strain MCT13 | 100.00% | OP087546 |
| TC-109-2 | *mucus bacterium* 10 | 99.17% | OP087547 |
| TC-111 | *Pseudomonas* sp. BBAPs-01 | 98.67% | OP087548 |
| TC-12 | *Pseudomonas stutzeri* strain OsEnb_ALM_B7 | 100.00% | OP087527 |
| TC-1-2 | *Pseudomonas stutzeri* | 99.72% | OP087578 |
| TC-123 | *Pseudomonas* sp. WXBSA | 98.42% | OP087550 |
| TC-123-1 | *mucus bacterium* 10 | 99.24% | OP087551 |
| TC-126 | *Ochrobactrum anthropi* strain CGKV-305c-OA | 99.71% | OP102540 |
| TC-127 | *mucus bacterium* 10 | 98.61% | OP087552 |
| TC-128 | *mucus bacterium* 10 | 99.38% | OP087553 |
| TC-13 | *Pseudomonas stutzeri* strain Os_Ep_PSA_22 | 100.00% | OP087528 |
| TC-132 | *Pseudomonas stutzeri* strain Z12 | 99.93% | OP087554 |
| TC-133 | *mucus bacterium* 10 | 99.51% | OP087555 |
| TC-137 | *Pseudomonas stutzeri* | 99.16% | OP087556 |
| TC-138 | uncultured bacterium | 99.65% | OP050460 |
| TC-139 | *Paenibacillus montaniterrae* | 99.71% | OP087557 |
| TC-140 | *Bacterium* | 99.72% | OP087558 |
| TC-142 | *mucus bacterium* 10 | 99.79% | OP087559 |
| TC-144R | *Bacillus* sp. | 99.86% | OP087560 |
| TC-146 | *Pseudomonas stutzeri* | 98.82% | OP087561 |
| TC-148 | *Pseudomonas stutzeri* strain SM12 | 99.86% | OP087562 |
| TC-151 | *Paenibacillus* sp. PF4D_4 | 99.71% | OP087563 |
| TC-152 | *Pseudomonas* sp. DGM UTI1a | 99.93% | OP087564 |
| TC-153 | *Pseudomonas stutzeri* | 99.79% | OP087565 |
| TC-157 | *Pseudomonas stutzeri* strain K-2-7 | 100.00% | OP087566 |
| TC-158 | *Agromyces* sp. | 100.00% | OP087567 |
| TC-159 | *Pseudomonas stutzeri* strain XX1 | 100.00% | OP087568 |
| TC-17 | *Agromyces indicus* strain TUN/POB/14 | 100.00% | OP087529 |
| TC-172 | *Pseudomonas* sp. WXBSA | 99.37% | OP087569 |
| TC-179 | *Paenibacillus montaniterrae* | 99.07% | OP087570 |
| TC-18 | *Brucella anthropi* | 100.00% | OP087530 |
| TC-180 | *Sphingomonas turrisvirgatae* | 99.14% | OP087571 |
| TC-185 | *Bacillus subtilis* | 99.86% | OP087572 |
| TC-189 | *Pseudomonas stutzeri* | 98.53% | OP087573 |
| TC-19 | *Bacterium* strain BP8 | 100.00% | OP087531 |
| TC-190 | *mucus bacterium* 10 | 99.93% | OP087574 |
| TC-193 | *Pseudomonas stutzeri* | 98.33% | OP087575 |
| TC-194 | *Bacillus* sp. JXR3 | 99.07% | OP087576 |
| TC-199 | *Pseudomonas stutzeri* | 98.61% | OP087577 |
| TC-22 | *Agromyces indicus*strain Mix6 | 100.00% | OP087532 |
| TC-23 | *Bacillus subtilis* | 100.00% | OP087533 |
| TC-27 | *Brevibacillus* sp. | 100.00% | OP087534 |
| TC-28 | *Pseudomonas stutzeri* strain GR 114 | 99.86% | OP087535 |
| TC-39-2 | *Pseudomonas putida* | 99.44% | OP087536 |
| TC-51 | *Ochrobactrum* sp. | 100.00% | OP102687 |
| TC-56B | *Pseudomonas* sp. strain DKF | 100.00% | OP087537 |
| TC-57 | *Bacterium* strain S76 | 100.00% | OP087538 |
| TC-58 | *Pseudomonas stutzeri*strain W12 | 100.00% | OP087539 |
| TC-59 | *Bacterium* strain S76 | 99.16% | OP087540 |
| TC-63 | *Agromyces indicus* strain NIO-1018 | 100.00% | OP087541 |
| TC-64 | uncultured *Aquiflexum* sp. | 97.19% | OP050457 |
| TC-66-1 | *Brucella anthropi* | 99.92% | OP087542 |
| TC-71 | *Pseudomonas stutzeri* strain Atecer4D | 99.93% | OP087543 |
| TC-81 | uncultured *Aquiflexum* sp. | 96.43% | OP050458 |
| TC-94 | uncultured *Lysobacter* sp. clone 1-I | 99.43% | OP050459 |
| TC-95 | *Bacillus safensis* | 99.31% | OP087544 |
| TC-R30-1 | *Pseudomonas stutzeri* strain 5-N-1 | 100.00% | OP076535 |
| TC-R36 | *Pseudomonas stutzeri* strain E141 | 100.00% | OP076540 |
| TC-R48 | *Pseudomonas stutzeri* strain SM12 | 100.00% | OP076543 |

**Tab S3. Direct plating methods obtained Strains**

| Name of Isolate | Closest related microorganism | Isolation culture media | 16S rRNA gene similarity (%) | GenBank accession number of isolates |
| --- | --- | --- | --- | --- |
| TC-TF1 | *Bacillus halodurans* strain XJU-4 | FW70 | 99.31% | ON573302 |
| TC-TR21 | *Agromyces indicus* strain NIO-1018 | R2A | 98.60% | ON573322 |
| TC-TR12 | *Agromyces indicus* strain TUN/POB/14 | R2A | 100.00% | ON573295 |
| TC-TR29 | *Bacillus clausii* strain XJU-2 | R2A | 97.75% | ON573358 |
| TC-TR23 | *Bacillus licheniformis* strain ROA205 | R2A | 99.86% | ON573326 |
| TC-TR2 | *Bacillus* sp. D1 | R2A | 99.45% | ON573321 |
| TC-TR28 | *Brevibacterium* sp. 2 | R2A | 99.58% | ON573357 |
| TC-TR5 | *Brevibacterium* sp. MN3-3 | R2A | 99.30% | ON573304 |
| TC-TR9 | *Brevibacterium* sp*.* MN3-3 | R2A | 99.58% | ON573303 |
| TC-TR15 | *Brevibacterium* sp. MN3-3 | R2A | 99.16% | ON573305 |
| TC-TR17 | *Brevibacterium* sp. MN3-3 | R2A | 99.02% | ON573307 |
| TC-TR20 | *Brevibacterium* sp. MN3-3 | R2A | 99.79% | ON573306 |
| TC-TR27 | *Brevibacterium* sp. MN3-3 | R2A | 99.65% | ON573315 |
| TC-TR8 | *Brevibacterium* sp. strain MGB 0544 | R2A | 99.24% | ON573319 |
| TC-TR24 | *Brevibacterium* sp. strain MGB 0544 | R2A | 98.61% | ON573355 |
| TC-TR3 | *Brevibacterium* sp. strain MX04 | R2A | 99.29% | ON573293 |
| TC-TR6 | *Brevibacterium* sp. strain ZCA-10 | R2A | 100.00% | ON573294 |
| TC-TR13 | *Brevibacterium* sp. strain ZCA-10 | R2A | 98.88% | ON573317 |
| TC-TR19 | *Geobacillus mahadia* strain Geo-05 | R2A | 99.59% | ON573323 |
| TC-TR1 | *Brevibacterium* sp.49 | R2A | 99.52% | ON573312 |
| TC-TR7 | *Geobacillus stearothermophilus* strain BGSC 9A21 | R2A | 99.25% | ON573318 |
| TC-TR17-2 | *Paenibacillus lautus* strain B1010/17 | R2A | 99.11% | ON573320 |
| TC-TR25 | *Sphingomonas olei* strain NM83_B4-11 | R2A | 98.00% | ON573353 |
| TC-TR26 | *Sphingomonas olei* strain NM83_B4-11 | R2A | 98.49% | ON573354 |
| TC-TR33 | *Sphingomonas* sp. 3.58 | R2A | 99.00% | ON573352 |
| TC-TR16 | *Sphingomonas* sp. strain HER1 | R2A | 100.00% | ON573300 |

**Tab S4 Screening of thermo-tolerant strains**

| Name of  Isolate | Closest related microorganism | Method | Withstand  temperature |
| --- | --- | --- | --- |
| TC-TR1 | *Brevibacterium* sp.49 | direct plating | 55℃ |
| TC-TR13 | *Brevibacterium* sp. strain ZCA-10 | direct plating | 55℃ |
| TC-TR15 | *Brevibacterium* sp. MN3-3 | direct plating | 55℃ |
| TC-TR16 | *Sphingomonas* sp. strain HER1 | direct plating | 65℃ |
| TC-TR17 | *Brevibacterium* sp. MN3-3 | direct plating | 75℃ |
| TC-TR17-2 | *Paenibacillus lautus* strain B1010/17 | direct plating | 55℃ |
| TC-TR19 | *Geobacillus mahadia* strain Geo-05 | direct plating | 55℃ |
| TC-TR2 | *Geobacillus stearothermophilus*  strain R2 | direct plating | 85℃ |
| TC-TR20 | *Brevibacterium* sp. MN3-3 | direct plating | 55℃ |
| TC-TR21 | *Agromyces indicus* strain NIO-1018 | direct plating | 85℃ |
| TC-TR23 | *Bacillus licheniformis* strain ROA205 | direct plating | 55℃ |
| TC-TR24 | *Brevibacterium* sp. strain MGB 0544 | direct plating | 65℃ |
| TC-TR25 | *Sphingomonas olei* strain NM83_B4-  11 | direct plating | 55℃ |
| TC-TR26 | *Sphingomonas olei* strain NM83_B4-  11 | direct plating | 55℃ |
| TC-TR27 | *Brevibacterium* sp. MN3-3 | direct plating | 55℃ |
| TC-TR28 | *Brevibacterium* sp. 2 | direct plating | 55℃ |
| TC-TR33 | *Sphingomonas* sp. 3.58 | direct plating | 55℃ |
| TC-TR5 | *Brevibacterium* sp. MN3-3 | direct plating | 55℃ |
| TC-TR6 | *Brevibacterium* sp. strain ZCA-10 | direct plating | 55℃ |
| TC-TR7 | *Geobacillus stearothermophilus*  strain BGSC 9A21 | direct plating | 65℃ |
| TC-TR9 | *Brevibacterium* sp*.* MN3-3 | direct plating | 55℃ |
| TC-106 | *Pseudomonas stutzeri* strain PST-03 | modified ichip | 45℃ |

| TC-107 | *Sphingomonas turrisvirgatae* strain  MCT13 | modified ichip | 45℃ |
| --- | --- | --- | --- |
| TC-1-2 | *Pseudomonas stutzeri* | modified ichip | 55℃ |
| TC-123 | *Pseudomonas* sp. WXBSA | modified ichip | 55℃ |
| TC-132 | *Pseudomonas stutzeri* strain Z12 | modified ichip | 65℃ |
| TC-144R | *Bacillus* sp. | modified ichip | 55℃ |
| TC-152 | *Pseudomonas* sp. DGM UTI1a | modified ichip | 45℃ |
| TC-153 | *Pseudomonas stutzeri* | modified ichip | 75℃ |
| TC-172 | *Pseudomonas* sp. WXBSA | modified ichip | 55℃ |
| TC-179 | *Paenibacillus montaniterrae* | modified ichip | 55℃ |
| TC-180 | *Sphingomonas turrisvirgatae* | modified ichip | 55℃ |
| TC-185 | *Bacillus subtilis* | modified ichip | 55℃ |
| TC-193 | *Pseudomonas stutzeri* | modified ichip | 55℃ |
| TC-194 | *Bacillus* sp. JXR3 | modified ichip | 55℃ |
| TC-199 | *Pseudomonas stutzeri* | modified ichip | 55℃ |
| TC-23 | *Bacillus subtilis* | modified ichip | 55℃ |
| TC-27 | *Brevibacillus* sp. | modified ichip | 55℃ |
| TC-28 | *Pseudomonas stutzeri* strain GR 114 | modified ichip | 45℃ |
| TC-56B | *Pseudomonas* sp. strain DKF | modified ichip | 55℃ |
| TC-94 | uncultured *Lysobacter* sp. clone 1-I | modified ichip | 45℃ |
| TC-95 | *Bacillus safensis* | modified ichip | 45℃ |
| TC-F10 | *Alkalihalobacillus halodurans C-125* | modified ichip | 85℃ |
| TC-FR12 | *uncultured Lysobacter* sp*.* | modified ichip | 65℃ |
| TC-FR12-2 | *Pseudomonas stutzeri* strain W12 | modified ichip | 45℃ |
| TC-FR13 | *Pseudomonas stutzeri* strain  WWvii23 | modified ichip | 55℃ |
| TC-FR15 | *Pseudomonas sp.* WXBSA | modified ichip | 55℃ |
| TC-FR2- | *uncultured Lysobacter* sp. | modified ichip | 55℃ |
| TC-FR20 | *Agromyces* sp*.* | modified ichip | 55℃ |

| TC-FR21 | *Pannonibacter phragmitetus* | modified ichip | 65℃ |
| --- | --- | --- | --- |
| TC-FR3 | *Pannonibacter* sp*.* | modified ichip | 55℃ |
| TC-FR4 | *Pseudomonas* sp*.* 2(2013) | modified ichip | 55℃ |
| TC-FR7 | *uncultured Lysobacter* sp. | modified ichip | 45℃ |
| TC-R(9)H | *Sphingomonas* sp*.* | modified ichip | 65℃ |
| TC-R1 | *uncultured Lysobacter* sp*.* | modified ichip | 85℃ |
| TC-R12 | *Bacillus halodurans* strain NS928R | modified ichip | 65℃ |
| TC-R13 | *Pseudomonas stutzeri* strain *QS169* | modified ichip | 65℃ |
| TC-R14 | *Pannonibacter* sp*.* strain DJ-1 | modified ichip | 45℃ |
| TC-R15 | *Bacillus* sp*.* | modified ichip | 55℃ |
| TC-R16 | *uncultured Lysobacter* sp*.* | modified ichip | 55℃ |
| TC-R19 | *Agromyces indicus* | modified ichip | 55℃ |
| TC-R20 | *uncultured Lysobacter* sp. | modified ichip | 85℃ |
| TC-R21 | *Pannonibacter* sp*.* | modified ichip | 65℃ |
| TC-R23 | *Bacillus* sp. CZB12 | modified ichip | 65℃ |
| TC-R24D | *Pannonibacter* sp*.* strain DJ-1 | modified ichip | 65℃ |
| TC-R2R | *uncultured Lysobacter* sp. | modified ichip | 55℃ |
| TC-R3 | *Agromyces indicus* | modified ichip | 55℃ |
| TC-R31- | *Agromyces indicus* | modified ichip | 85℃ |
| TC-R33 | Bacterium strain BMV5 | modified ichip | 65℃ |
| TC-R33-1 | Bacterium strain BLEC9.3 | modified ichip | 55℃ |
| TC-R36 | *Pseudomonas stutzeri* strain E141 | modified ichip | 65℃ |
| TC-R4R | *Pseudomonas stutzeri* strain FN9 | modified ichip | 65℃ |
| TC-R57-2 | *Agromyces indicus* | modified ichip | 55℃ |
| TC-R6 | *Microbacterium aurantiacum* | modified ichip | 55℃ |
| TC-TF1 | *Bacillus halodurans* strain XJU-4 | modified ichip | 45℃ |
| TC-TR29 | *Bacillus clausii* strain XJU-2 | modified ichip | 45℃ |
